# Different regression equations relate age to the incidence of Lauren types 1 and 2 stomach cancer in the SEER database: these equations are unaffected by sex or race

**DOI:** 10.1186/1471-2407-6-65

**Published:** 2006-03-15

**Authors:** Mitchell S Wachtel, Yan Zhang, Maurizio Chiriva-Internati, Eldo E Frezza

**Affiliations:** 1 Department of Pathology, Texas Tech University Health Sciences Center, Lubbock, USA; 2 Department of Family and Community Medicine, Texas Tech University Health Sciences Center, Lubbock, USA; 3 Department of Surgery, Texas Tech University Health Sciences Center, Lubbock, USA

## Abstract

**Background:**

Although impacts upon gastric cancer incidence of race, age, sex, and Lauren type have been individually explored, neither their importance when evaluated together nor the presence or absence of interactions among them have not been fully described.

**Methods:**

This study, derived from SEER (Surveillance, Epidemiology, and End Results (SEER) Program of the National Cancer Institute) data, analyzed the incidences of gastric cancer between the years 1992–2001. There were 7882 patients who had developed gastric cancer. The total denominator population was 145,155, 669 persons (68,395,787 for 1992–1996, 78,759,882 for 1997–2001). Patients with multiple tumors were evaluated as per the default of the SEER*Stat program. 160 age-, five year period (1992–1996 vs 1997–2001)-, sex-, race (Asian vs non-Asian)-, Lauren type- specific incidences were derived to form the stratified sample evaluated by linear regression. (160 groups = 2 five year periods × 2 race groups × 2 sexes × 2 Lauren types × 10 age groups.) Linear regression was used to analyze the importance of each of these explanatory variables and to see if there were interactions among the explanatory variables.

**Results:**

Race, sex, age group, and Lauren type were found to be important explanatory variables, as were interactions between Lauren type and each of the other important explanatory variables. In the final model, the contribution of each explanatory variable was highly statistically significant (t > 5, d.f. 151, P < 0.00001). The regression equation for Lauren type 1 had different coefficients for the explanatory variables Race, Sex, and Age, than did the regression equation for Lauren type 2.

**Conclusion:**

The change of the incidence of stomach cancer with respect to age for Lauren type 1 stomach cancer differs from that for Lauren type 2 stomach cancers. The relationships between age and Lauren type do not differ across gender or race. The results support the notion that Lauren type 1 and Lauren type 2 gastric cancers have different etiologies and different patterns of progression from pre-cancer to cancer. The results should be validated by evaluation of other databases.

## Background

Worldwide, the stomach is the second most common site of origin of cancer [1]. Although an array of histologic classifications is in use, that proposed by Lauren [2], retains its durability because its two types have been the most widely compared and because pathologists can reproducibly distinguish Lauren type 1 from Lauren type 2 cancers [3]. Year of diagnosis [4-15], gender [5,12,16-25], race [22,26-33], age [10,13,14,16,18,19,21,24,26,35-38], and Lauren type [5,11,18,19,26,27,35,39-41], have all been found by recent epidemiologic studies to be important explanatory variables for stomach cancer incidence; various interactions among these variables have also been demonstrated [5,10-14,16,19,22,24,35]. We hypothesized that, by examining a large database, it might be possible to evaluate each of the above factors and interactions among the factors to explain differences in stomach cancer incidence.

The Surveillance, Epidemiology, and End Results (SEER) Program of the National Cancer Institute is an authoritative source of information on cancer incidence and survival in the United States that currently collects and publishes cancer incidence and survival data from 14 population-based cancer registries and three supplemental registries covering approximately 26 percent of the US population; the SEER website provides extensive information about it [42].

The study used SEER to evaluate the contributions of age, sex, race (Asian vs non-Asian), year of diagnosis (1992–1996 vs 1997–2001), and Lauren type to gastric cancer incidence. The study showed Lauren type 1 tumor incidence increased with respect to age in a different way than did Lauren type 2 tumor incidence; the regression equations that described these relations were the same for men and women and for Asians and non-Asians. Incidence was considered in terms of the natural logarithms of the rates of development, over a five year period, of stomach cancer.

## Methods

### Data acquisition

The SEER data base, SEER 11 Regs + AK Public-Use, Nov 2003 Sub for Expanded Races (1992–2001) was used [44]. The analysis was limited to persons with stomach cancer 40 years and older. The SEER*STAT program stratified persons who developed stomach cancer and the underlying population from which they emanated, into ten age groups, two sexes, two races, two Lauren types, and two five year periods. This produced two numbers for each of 160 age, race, sex, Lauren type, and five year period groups, a number of persons who developed stomach cancer and an underlying number of persons in the denominator. Lauren type 1 was defined as those patients whose cancers showed intestinal morphology (M-8144); Lauren type 2 was defined as those patients whose cancers showed diffuse (M-8145), signet ring cell (M-8490), or linitis plastica (M-8142) morphology. The ten age groups were defined as follows: 40–44 (group 1), 45–49 (group 2), 50–54 (group 3), 55–59 (group 4), 60–64 (group 5), 65–69 (group 6), 70–74 (group 7), 75–79 (group 8), 80–84 (group 9), and 85+ (group 10). The relative lack of blacks who had stomach cancer meant many analyzed cells would be zero, making the analysis suspect. The two races evaluated were Asian and non-Asian. Persons of unknown race were excluded. Multiple primaries were handled by the default set by the SEER*STAT program. The two five-year periods comprised the years 1992–1996 and 1997–2001. Table 1 schematizes the data acquisition.

Hence, SEER generated 160 numbers pairs. Each pair comprised a number of persons who developed stomach cancer and a number of persons in the denominator. For each set a rate was calculated by dividing the number of persons with stomach cancer by the number of persons in the denominator. For example, from 1992–1996, 35 Lauren type 1 stomach cancers were observed among 2,752,873 non-Asian men ages 40–44: the rate was 1.27 × 10^-5^. From 1997–2001, of 366,766 Asian women aged 65–69, 55 developed Lauren type 1 gastric cancer: the rate was 1.60 × 10^-4^.

## Statistical methods

### Software

R was used for data analysis.

#### Data transformation

Counts and population provided by SEER*STAT were used to calculate rates. Preliminary data analysis showed the distribution of rates lacked a normal distribution. One cell had no persons with cancer; to take the natural logarithms of rates in a circumstance in which a zero cell is present, one may increase for all cells the numerator and the denominator by 0.5 [44]:

ln(ca) = ln [(persons with cancer + 0.5)/(persons in denominator + 0.5)]

### Model selection

All models tested were linear regression models with the response variable being the logarithms of the cancer rates, as defined above. Analysis of variance determined which model best reflected the data. Covariates were always added to regression; the ratio of the change in the residual sum of squares (ΔRSS) to the RSS before the covariate was added was compared with an F test. Independent (explanatory or predictor) variables included: A) Five year period (1992–1996 = 0, 1997–2001 = 1), B) Sex (men = 0, women = 1), C) Race (Non Asian = 0, Asian = 1), D) Lauren type (type 1 = 0, type 2 = 1), E) Age Group (40–44 = 1, 45–49 = 2, 50–54 = 3, etc.). All ten possible two-way variable interactions were assessed. The null hypothesis was rejected if *P *< 0.05. Neither higher-order explanatory variable interactions nor non-linear relationships of explanatory variables to the response variable were considered in this analysis.

Evaluation of the data precluded the performance of Poisson regression: the mean number of patients with cancer was 49; the variance was 2224. When Poisson regression was tried with population as an offset, with or without the zero cell, all five potential explanatory variables (five year period, sex, race, Lauren type, and age) and all ten potential first order interactions were associated with the counts; each explanatory variable had an associated *z *> 600, *P *< 1 × 10^-10^. A residual plot showed the model lacked a good fit.

### Model adequacy

Standardized residuals were calculated by R. To test the assumption that the standardized residuals were normally distributed, a Shapiro-Wilks test was performed. To test the assumption that the mean of the standardized residuals was 0, a t-test was performed. To test the assumption that the standardized residuals had constant variance with respect to the fitted values, the standardized residuals were divided into quartiles and Bartlett's test for homogeneity of variances was performed. A data point was considered an outlier if its studentized residual, calculated by R, was greater than 3; dffits (a measure which gives greater weight to outlying observations) and Cook's distance (a measure of impact of the respective case on the regression equation), calculated by R were used to assess leverage; outliers that are found to have high leverage, large dffits and/or Cook's distances, are considered bad leverage points and are removed from the analysis.

## Results

### Raw data

The distribution of persons who developed cancer, stratified by Lauren type, five year period, sex, and race, is displayed in Table 2. The distribution of the denominator population, stratified by five year period, sex, and race, is shown in Table 3. Age group distributions for the persons who developed cancer and the denominator population are displayed in Table 4.

### Summary of model building

Initial evaluation showed rates lacked a normal distribution (Shapiro-Wilks W = 0.76, P < 0.0001). As discussed in the model adequacy section, the use of the logarithms of the rates yielded a model that fulfilled the assumptions of linear regression, once an outlier was removed; the residuals of that model did not prove to lack a normal distribution, did not have a mean that proved to differ from zero, and did not prove to lack homogeneity of variance. Table 5 displays the results of the univariate analyses. Significant associations between the natural logarithms of cancer rates and age group, race, Lauren type, and sex, but not five year period, were identified. Table 6 displays the results, with and without the outlier identified at model adequacy assessment, of ANOVA comparisons of sequential models; the results confirmed those of the univariate analyses. Table 7 displays the results of the analysis of interaction covariates. ANOVA demonstrated significant decreases in residual sum of squares when interactions of Lauren type and age group, Lauren type and race, and Lauren type and sex were added to regression. No other first order interaction was shown by ANOVA to decrease RSS sufficiently to reject the null hypothesis.

### Final model

The final model did not include five year period as an explanatory variable because 1) there was no association between the natural logarithms of the cancer rates and the five year period and 2) there was no demonstrated interaction between five year period and any other explanatory variable. The final model, displayed in Table 8, included the four other main effects (ME) covariates and the four interaction covariates found to significantly reduce residual sum of squares (RSS). Lauren type interacted with the group of explanatory variables that include Race, Age, and Sex, which did not interact with one another. Hence, Lauren type 1 and type 2 cancers differ as regards the shapes of the age distributions, sex, and race. When Lauren type is taken into account, similar statements cannot be made for differently-aged patients, for men and women, for the five year periods 1992–1996 and 1997–2001, and for Asians and non-Asians.

Two regression equations, using the values for the final model without the outlier, express the results:

Lauren type 1

ln(ca) = -14.15 - 1.07 × sex + 1.74 × race + 0.52 × age group

Lauren type 2

ln(ca) = -11.55 - 0.23 × sex + 0.97 × race + 0.28 × age group

For the above equations:

• ln(ca) is the response variable, the natural logarithm of the stomach cancer rates.

• Sex, Race, and Age are explanatory variables (sometimes called *risk factors*)

• The numbers in front of the explanatory variables are called regression *coefficients *or *beta weights*. Coefficients are interpreted as follows: if Sex and Age are held constant (or "controlling for" Sex and Age), then, for Lauren type 1 stomach cancer, mean ln(ca) increases by about 1.74 if the group is Asian; for Lauren type 2 stomach cancer, the corresponding increase is 0.97.

**Figure 1:**
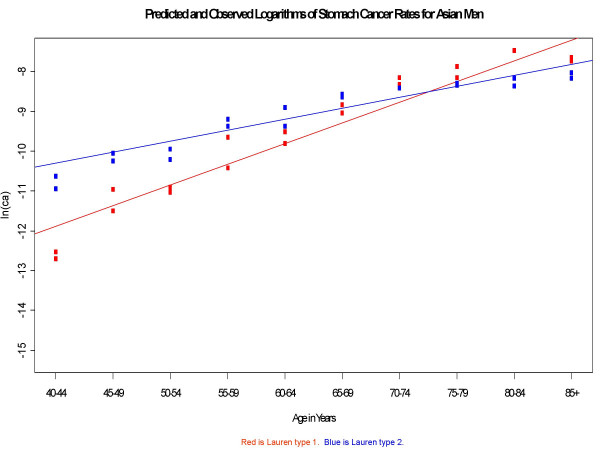
Plot of the natural logarithms of cancer rates, denoted as ln(ca), as a function of age in years for Asian men. Red references Lauren type 1 gastric cancer. Blue references Lauren type 2 gastric cancer. Lines represent predicted values. [see Additional file 1]

**Figure 2:**
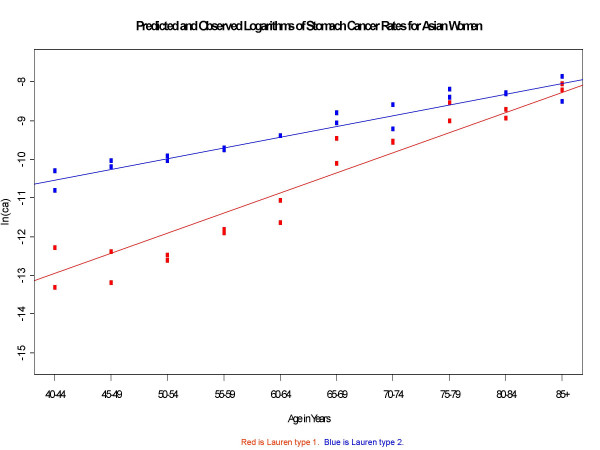
Plot of the natural logarithms of cancer rates, denoted as ln(ca), as a function of age in years for Asian women. Red references Lauren type 1 gastric cancer. Blue references Lauren type 2 gastric cancer. Lines represent predicted values. [see Additional file 1]

**Figure 3:**
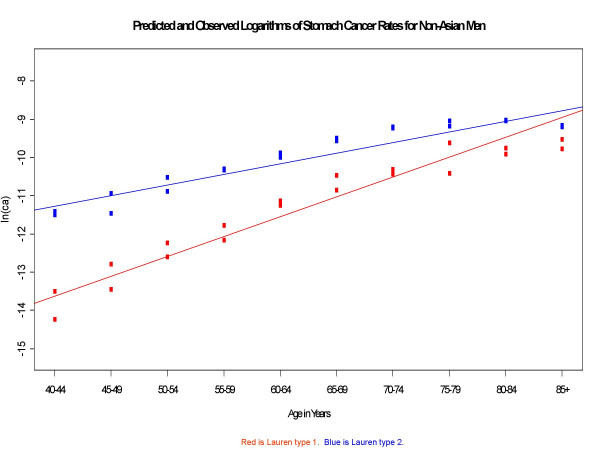
Plot of the natural logarithms of cancer rates, denoted as ln(ca), as a function of age in years for non-Asian men. Red references Lauren type 1 gastric cancer. Blue references Lauren type 2 gastric cancer. Lines represent predicted values. [see Additional file 1]

**Figure 4:**
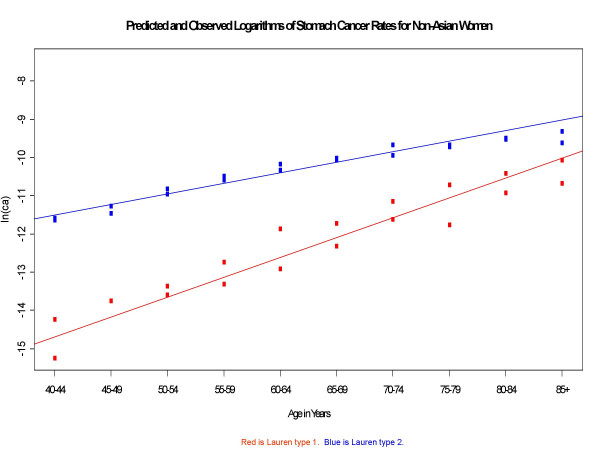
Plot of the natural logarithms of cancer rates, denoted as ln(ca), as a function of age in years for non-Asian women. Red references Lauren type 1 gastric cancer. Blue references Lauren type 2 gastric cancer. Lines represent predicted values. [see Additional file 1]

Figures 1, 2, 3 and 4 plot predicted and observed logarithms of stomach cancer rates versus age comparing Asian and non-Asian men and Asian and non-Asian women. Actual data confirms the regression equations: data points pretty closely approximate the lines in all cases. Although the races and sexes may start off with different incidences at age 40, changes in incidence with respect to age are quite similar. Because the incidence of patients with Lauren type 1 tumors 1) starts out lower than and 2) rises at almost twice the rate of the incidence of patients with Lauren type 2 tumors, the ratio of Lauren type 1 to type 2 incidences strongly depends on age. Compare Asian and non-Asian men: at age 40 both groups show a greater incidence of patients who developed Lauren type 2 tumors; at age 85 Asians show a greater incidence of patients who developed Lauren type 1 tumors, which is not true for non-Asians.

### Model adequacy

There was only one outlier, a data point with a studentized residual over 3. No non-Asian women, aged 45–49 years, developed Lauren type 1 stomach cancer; it was the only zero cell. The studentized residual, -5.40, corresponded with the largest Cook's distance, 0.183, and the dffit, -1.316, with the largest absolute value. The outlier was a bad leverage point. Although results for the outlier are displayed in the tables, the high leverage meant that the outlier should be excluded from the final analysis. Removing the outlier yielded the same choice of covariates.

Quantitative assessments of model adequacy are displayed in Table 9. The final model with the outlier had a set of standardized residuals with non-constant variance, when evaluated by Bartlett's test; the model without the outlier did not show this failure of model assumption. The final model with the outlier had a set of standardized residuals without a normal distribution, when evaluated by the Shapiro-Wilks test; the model without the outlier did not show this failure of model assumption. Neither the model with nor the model without the outlier had standardized residuals whose means differed from zero, when evaluated by a t test. Variable inflation factor analysis showed no problems with multicollinearity among the main explanatory variables.

## Discussion

This study found, in the SEER database, that Lauren type 1 and Lauren type 2 stomach cancers differ to such a degree that different regression equations are required to explain variations in their incidences. Sex, race (Asian or non-Asian), and age are explanatory variables, but the equations that relate these explanatory variables to the incidence of each Lauren type differ. Recent epidemiologic studies well support the rationale of the current study, namely to evaluate year of diagnosis (in this case five year period), sex, race, age and Lauren type. The articles also support the need for evaluation of interactions and also provide interesting thoughts about the limitations of administrative databases and other factors that should be considered in future studies.

### Year of diagnosis

Boyle [4] found stomach cancer in general was declining in incidence in Europe, as did Faycal [5], Pineros [6] in Columbia, Ardanaz [7] in Navarro, and Stracci [8] and Crocetti [9] in Italy. Liu's [10] results showed that over time, for both sexes, there were different trends for stomach cancer depending on the third of the stomach involved and the age of the patient. Henson [11] studied Lauren type 2 and Lauren type 1 incidences over time, revealing that the changes over time differed between the two Lauren types. Sunny's [12] study of Indian men and women revealed different rates of decline in stomach cancer, demonstrating an interaction of time of diagnosis and sex. Levi [13], using joinpoint regression analysis, found that the fall over time in gastric cancer rates was proportionally greater for older than for younger persons, although all showed a decline. The results of the study of Kobayashi [14], by contrast, gave the lion share of the decrement in gastric cancer for the young. For the Greenland Inuit, stomach cancer rates appear to have increased [15]. This study of SEER data did not show time of diagnosis or an interaction of any factor and time of diagnosis to be an important explanatory variable for the incidence of stomach cancer over the decade 1992–2001 in the United States.

### Sex

Marmo [16], Turkdogan [17], Faycal [5], Bani-Hanu [21], and Dobru [18] all showed men at greater risk for stomach cancer than women. Among Epstein Barr Virus positive cancers, the gender difference exists, but is only statistically significant for Lauren type 2 gastric cancers [19]. A family history of stomach cancer would appear to place women, but not men at increased risk [20]. Alaskan Native American men differ less from other American men than do Alaskan Native American women, demonstrating an interaction of sex and race [22]. Green tea consumption appears to protect women, but not men, from gastric cancer [23]. Japanese men appear to have a greater increase in risk as they age than do Japanese women [24]. Khan [25] found that different foods for men than for women increased the risk of gastric cancer. This study of SEER data identified gender as an important explanatory variable for the incidence of stomach cancer.

### Race

Ciliated metaplasia, a precursor to stomach cancer, occurs at different rates in the Pacific and Atlantic basins [26]. Yao [27] showed that Hispanics with stomach cancer differed in age than other persons and that Asians differed in survival than other persons. Multiple studies have placed Asians at greater risk of gastric cancer [28-30]. When a known risk factor, such as H. pylori, becomes universally acquired, it ceases to be a risk factor; this has been shown to have occurred in Koirea [31]. This study of SEER data did not find an interaction between race and sex, but did identify race as an explanatory variable for stomach cancer incidence.

As to black race, some have suggested that Caucasians are more likely than blacks to develop gastric cancer that arises in the cardia and that blacks are more likely than Caucasians to develop gastric cancer that arises outside the cardia [32,33]. There were insufficient patients in this study to subdivide the analysis by site within the stomach or to separately analyze black persons. To evaluate the importance of black race, studies would need 1) to have more black patients and 2) to take into account whether the cancer originated in the gastric cardia or not. The location of origin would be of interest in itself as immunohistochemical patterns of cardia and non-cardia gastric cancers differ [34].

### Age

Older persons more likely develop ciliated metaplasia than do young persons [26]. Multiple studies have shown that age is a vitally important factor to consider as regards the risk of stomach cancer [18,35,21]. In terms of other risk factors, there is good reason to think that H pylori's effect declines with age [36] and that acquisition of H pylori after age 1 may be less important in carcinogenesis than is acquisition before age 1 [37]. Marmo [16] and Tanaka [24] demonstrated an interaction of age and sex. Levi [13], Kobayashi [14], and Liu [10] showed an interaction of age and time of diagnosis. A prior study [38] suggested that the effect of environmental carcinogens is largely limited to childhood. The latter is in accord with our study, which would suggest that a person's risk of cancer is set at or below the age of 40 and that its expression occurs at predictable increments thereafter. This study of SEER data found age to be an explanatory variable for stomach cancer incidence.

### Lauren type

Loss of CDX2 may represent a marker of tumor progression in early gastric cancer and carcinomas with an intestinal, but not a non-intestinal phenotype [39]. The frequency of ciliated metaplasia differs between intestinal and non-intestinal stomach cancer types [26]. For some nations, Lauren type 1 cancer was more common than Lauren type 2 [18,40]. Yao [27] showed Hispanics differed in Lauren type from other persons. An interaction with between time of diagnosis and Lauren type exists [5,11]. van Beek [41] found that Epstein Barr Virus associated cancer was more frequently associated with Lauren type 1 than with Lauren type 2 adenocarcinoma. This study of SEER data found interactions of Lauren type and race, age, and gender to be of such importance that two different regression equations had to be created to describe the data. The importance of separating Lauren types from one another lies in part in the demonstrated multiple interactions between Lauren type and so many other variables, both those found in this study and those found in recent epidemiologic studies.

The above discourse allows one to appreciate the limitations and utility of the study. SEER is, like many of the sources of the other studies, an administrative database. Administrative databases lack a review of histopathology; the added loss of precision is unavoidable because such a review would increase the expense of any such study and decrease participation by hospitals, largely invalidating its results. As expected, specific program codes are not available on line for investigators, reviewers, readers, and editors to explore issues that may be important to them, such as the means of creation of a denominator in rate calculations. The website is excellent, but might also include readily accessible links to the data registries themselves and their policies and procedures, so investigators, reviewers, editors, and readers can satisfy any questions they might have as to such matters as data collection or the particular manner of dealing with multiple primaries for a particular study. No administrative database can keep a record of such things as H pylori rates, genetic markers, food intake, or any of the other above miscellaneous factors identified. As with any study, the number of factors that can be evaluated is limited both for reasons of data collection and for statistical reasons having to do with sample size; for this reason, a global explanation encompassing all potential factors cannot be expected. The most any epidemiologic study can offer is a partial explanation of complex phenomena. Most vital, the above referenced recent studies show that any conclusion derived by examination of a particular population must be verified by evaluation of multiple populations. This is because factors that are important in one population may be unimportant in another population; only by repeating an analysis in multiple populations can an epidemiologic conclusion be considered verified. Notwithstanding these caveats, such studies of epidemiology have great practical significance; Marmo [16] used such results to design a screening protocol for stomach cancer based on age and sex so as to reduce cost.

## Conclusion

In summary, two regression equations were derived from the SEER database to explain differences in stomach cancer incidence, one for Lauren type 1 stomach cancers, one for Lauren type 2 stomach cancers. Each regression equation revealed a simple relationship between the natural logarithm of stomach cancer incidence rates and age. These equations were the same for men and women and for Asians and non-Asians. These results should be verified by similar evaluations conducted in other populations.

## Competing interests

The author(s) declare that they have no competing interests.

## Authors' contributions

MW and YZ performed the regression analysis. MC, MW, and EF conceived of the study, and participated in its design and coordination and helped to draft the manuscript. All authors read and approved the final manuscript.

## Pre-publication history

The pre-publication history for this paper can be accessed here:



## Figures and Tables

**Table 1:** Schema for acquisition of data from SEER for this study.

**DATA **	
	
**STATISTIC **	
Statistic: Crude Rates	
	
**SELECTION **	
Case Only: {Site and Morphology.Site recode} = 'Stomach'	
Option: Select only malignant behaviour	
	
**TABLE **	
Row: Year of dx, 92–96, 97–01 [Year of diagnosis]	
	Men and Women [Sex]
	Stomach cancer types [Histologic Type ICD-O-3]
	Race, Asian or not, no unknown [Race recode Y]
Column: Age recode with <1 year olds	
	
**USER DEFINITIONS **	
	Stomach cancer types [Histologic Type ICD-O-3]
	intestinal = 8144
	non-intestinal = 8142,8145,8490
	Year of dx, 92–96, 97–01 [Year of diagnosis]
	1992–1996 = 1992, 1993, 1994, 1995, 1996
	1997–2001 = 1997, 1998, 1999, 2000, 2001
	Sex [Sex]
	Male = Male
	Female = Female
	Race, Asian or not, no unknown [Race recode Y]
	Asian = Asian or Pacific Islander
	Non-Asian = All other except unknown

**Table 2:** Frequency distribution of 7882 persons who developed stomach cancer, by five year period, by sex, by race, and by Lauren type.

five year period	1992–1996	1997–2001
	3,429	4,453
Sex	Men	women
	4,399	3,483
Race	Asian	non-Asian
	2,059	5,823
Lauren type	type 1	Type 2
	1,992	5,890

**Table 3:** Frequency distribution of denominator population by five year period, by sex, and by race.

five year period	1992–1996	1997–2001
	68,395,787	76,759,882
Sex	men	women
	67,831,186	77,324,483
Race	Asian	non-Asian
	14,600,067	130,555,602

**Table 4:** Frequency distribution of persons with cancer and persons in denominator by age group.

Ages	Persons with Cancer		Persons in Denominator	
40–44	353	4.5%	29,331,208	20.2%
45–49	442	5.6%	25,218,817	17.4%
50–54	542	6.9%	20,412,681	14.1%
55–59	631	8.0%	15,655,389	10.8%
60–64	762	9.7%	13,042,756	9.0%
65–69	1,056	13.4%	11,913,627	8.2%
70–74	1,250	15.9%	10,623,230	7.3%
75–79	1,203	15.3%	8,522,907	5.9%
80–84	874	11.1%	5,660,379	3.9%
85+	769	9.8%	4,774,675	3.3%
Total	7,882	100.0%	145,155,669	100.0%

**Table 5:** Univariate regression of the natural logarithm of the rate of stomach cancer on five year period, on sex, on race, on Lauren type, and on age group.

Explanatory variable	Intercept	R^2^	Estimate	Std Error	t	Pr (> | t|)
five year period	-10.40	0.002	0.16	0.26	0.61	0.54
Sex	-9.98	0.042	-0.68	0.26	-2.62	0.01
Race	-11.01	0.174	1.38	0.24	5.77	4.1 × 10^-8^
Lauren type	-10.98	0.158	1.31	0.24	5.44	2.0 × 10^-7^
age group	-12.54	0.491	0.40	0.03	12.35	< 1 × 10 ^-10^

**Table 6:** Comparisons of linear regression models, with and without outlier, of the natural logarithm of the rate of stomach cancer on the main explanatory variables. The difference between the residual sum of squares (RSS) before and after each explanatory variable had been added to regression (ΔRSS) was divided by RSS and multiplied by the error df to yield F, whose numerator df was 1 and denominator df was the error df.

WITHOUT OUTLIER					
Model Covariates	RSS	ΔRSS	error df	F	*P*

Null	403.26				
five year period	402.96	0.30	157	0.1	0.73
five year period + sex	388.32	14.64	156	5.9	0.02
five year period +sex + race	319.23	69.09	155	33.5	3.8 × 10^-8^
five year period + sex + race + Lauren type	255.93	63.30	154	38.1	5.7 × 10^-9^
five year period + sex + race + Lauren type + age	50.64	205.29	153	620.2	< 1 × 10 ^-10^
					
WITH OUTLIER					
Model Covariates	RSS	ΔRSS	error df	F	*P*
Null	437.81				
five year period	436.79	1.02	158	0.4	0.54
five year period + sex	418.50	18.29	157	6.9	0.01
five year period +sex + race	342.38	76.12	156	34.7	2.3 × 10^-8^
five year period + sex + race + Lauren type	273.23	69.15	155	39.2	3.5 × 10^-9^
five year period + sex + race + Lauren type + age	58.14	215.10	154	569.8	< 1 × 10 ^-10^

**Table 7:** Comparisons of linear regression models, with and without outlier, of the natural logarithm of the rate of stomach cancer on the main explanatory variables and each of ten interaction variables. The main effects (ME) comprised the explanatory variables five year period, sex, race, Lauren type, and age. The difference between the residual sum of squares (RSS) before and after the addition of each interaction variable to ME (ΔRSS) was divided by RSS and multiplied by error d.f. to yield F, whose numerator df was 1 and denominator df was error df.

WITHOUT OUTLIER					
Model Covariates	RSS	ΔRSS	error df	F	*P*

ME	50.64				
ME + five year period:sex	50.60	0.05	152	0.1	0.71
ME + five year period:race	50.09	0.55	152	1.7	0.20
ME + five year period: Lauren type	50.51	0.13	152	0.4	0.53
ME + five year period:age	50.04	0.60	152	1.8	0.18
ME + sex:race	50.62	0.02	152	0.1	0.82
ME + sex:Lauren type	43.99	6.65	152	23.0	3.9 × 10^-6^
ME + sex:age	50.63	0.02	152	0.0	0.83
ME + race:Lauren type	45.00	5.64	152	19.0	2.3 × 10^-5^
ME + race:age	49.50	1.14	152	3.5	0.06
ME + Lauren type:age	31.62	19.03	152	91.5	< 1 × 10 ^-10^
					
WITH OUTLIER					
Model Covariates	RSS	ΔRSS	error df	F	*P*
ME	58.14				
ME + five year period:sex	57.95	0.19	153	0.5	0.48
ME + five year period:race	57.22	0.92	153	2.5	0.12
ME + five year period: Lauren type	57.80	0.34	153	0.9	0.35
ME + five year period:age	57.88	0.25	153	0.7	0.41
ME + sex:race	58.01	0.13	153	0.3	0.57
ME + sex:Lauren type	50.34	7.79	153	23.7	2.8 × 10^-6^
ME + sex:age	58.11	0.02	153	0.1	0.81
ME + race:Lauren type	51.44	6.70	153	19.9	1.6 × 10^-5^
ME + race:age	57.50	0.63	153	1.7	0.20
ME + Lauren type:age	36.88	21.25	153	88.2	< 1 × 10 ^-10^

**Table 8:** Final multiple linear regression models, with and without outlier, of the natural logarithm of the rate of stomach cancer.

WITHOUT OUTLIER						
Parameter	Estimate	Std Error	t	df	Pr(>| t|)	95% Conf Int

Intercept	-14.15	0.11	-134.44	151	< 1 × 10 ^-10^	-14.35 – -13.94
Sex	-1.07	0.08	-13.15	151	< 1 × 10 ^-10^	-1.23 – -0.91
Race	1.74	0.08	21.42	151	< 1 × 10 ^-10^	1.58 – 1.90
Lauren type	2.59	0.15	17.53	151	< 1 × 10 ^-10^	2.30 – 2.89
Age	0.52	0.01	36.69	151	< 1 × 10 ^-10^	0.49 – 0.55
age:Lauren type	-0.24	0.02	-12.19	151	< 1 × 10 ^-10^	-0.28 – -0.20
race:Lauren type	-0.77	0.11	-6.72	151	3.6 × 10 ^-10^	-0.99 – -0.54
sex:Lauren type	0.83	0.11	7.28	151	< 1 × 10 ^-10^	0.61 – 1.06
						
Overall Model	Std Error	R^2^	F	df num	df den	*P*
0.36	0.95	421	7	151	< 1 × 10 ^-10^	
						
WITH OUTLIER						
Parameter	Estimate	Std Error	t	df	Pr(>| t|)	95% Conf Int
Intercept	-14.23	0.11	-125.58	152	< 1 × 10 ^-10^	-14.45 – -14.01
Sex	-1.12	0.09	-12.73	152	< 1 × 10 ^-10^	-1.29 – -0.94
Race	1.79	0.09	20.38	152	< 1 × 10 ^-10^	1.62 – 1.96
Lauren type	2.68	0.16	16.71	152	< 1 × 10 ^-10^	2.36 – 2.99
Age	0.53	0.02	34.72	152	< 1 × 10 ^-10^	0.50 – 0.56
age:Lauren type	-0.25	0.02	-11.74	152	< 1 × 10 ^-10^	-0.30 – -0.21
race:Lauren type	-0.82	0.12	-6.59	152	6.7 × 10 ^-10^	-1.06 – -0.57
sex:Lauren type	0.88	0.12	7.11	152	< 1 × 10 ^-10^	0.64 – 1.13
						
Overall Model	Std Error	R^2^	F	df num	df den	*P*
0.39	0.95	384	7	152	< 1 × 10 ^-10^	

**Table 9:** Quantitative assessments of model adequacy.

Shapiro-Wilks normality test performed on standardized residuals.				
	*W*	*P*		

				
Without outlier	0.984	0.06		
With outlier	0.960	0.0001		
T test to see if mean of standardized residuals was not zero.				
	Mean	T	df	*P*
Without outlier	-0.003	-0.03	158	0.97
With outlier	-0.003	-0.03	159	0.97
				
Bartlett's test to see if the standardized residuals, divided into four groups by their corresponding fitted values, lacked constant variances.				
	K^2^	Df	*P*	
Without outlier	4.43	3	0.22	
With outlier	15.02	3	0.002	
